# Gut microbiota, circulating cytokines and dementia: a Mendelian randomization study

**DOI:** 10.1186/s12974-023-02999-0

**Published:** 2024-01-04

**Authors:** Dong Ji, Wen-Zhu Chen, Lei Zhang, Zhi-Hua Zhang, Li-Jian Chen

**Affiliations:** 1 Department of Anesthesiology, The First Affiliated Hospital of Anhui Medical University, No.218 Jixi Road, Shushan District, Hefei, Anhui China; 2 Department of Epidemiology and Biostatistics, School of Public Health, Anhui Medical University, Hefei, Anhui China

**Keywords:** Gut microbiota, Dementia, Cytokines, Mendelian randomization

## Abstract

**Background:**

Some studies have shown that gut microbiota may be associated with dementia. However, the causal effects between gut microbiota and different types of dementia and whether cytokines act as a mediator remain unclear.

**Methods:**

Gut microbiota, cytokines, and five dementia types, including Alzheimer’s disease (AD), frontotemporal dementia (FTD), dementia with Lewy body (DLB), vascular dementia (VD), and Parkinson’s disease dementia (PDD) were identified from large-scale genome-wide association studies (GWAS) summary data. We used Mendelian randomization (MR) to investigate the causal relationships between gut microbiota, cytokines, and five types of dementia. Inverse variance weighting (IVW) was used as the main statistical method. In addition, we explored whether cytokines act as a mediating factor in the pathway from gut microbiota to dementia.

**Results:**

There were 20 positive and 16 negative causal effects between genetic liability in the gut microbiota and dementia. Also, there were five positive and four negative causal effects between cytokines and dementias. Cytokines did not act as mediating factors.

**Conclusions:**

Gut microbiota and cytokines were causally associated with five types of dementia, and cytokines seemed not to be the mediating factors in the pathway from gut microbiota to dementia.

**Supplementary Information:**

The online version contains supplementary material available at 10.1186/s12974-023-02999-0.

## Introduction

Dementia is a syndrome characterized by cognitive and memory impairment. Its subtypes are Alzheimer’s disease (AD), frontotemporal dementia (FTD), dementia with Lewy body (DLB), and vascular dementia (VD) [1]. In addition, the full spectrum of cognitive impairment occurs in individuals with Parkinson’s disease (PD), from subjective cognitive decline and mild cognitive impairment to Parkinson’s disease dementia (PDD) [2].

The ‘gut microbiota’ can be defined as all the species within the ecosystem and are considered the largest reservoir of microbes in the human body, containing about 10^14^ microbes [3]. The human gut contains approximately 1.5 kg of cells, comprising Archaea and Eukaryotes, but are predominantly bacterial [4]. These gastrointestinal microbiotas play important roles in physiological homeostasis and metabolism, including immune system development, vitamin production, and nutrient absorption [5]. Studies have shown that gut microbiotas were associated with dementia [3, 6]. In addition, gut microbiota plays an important role in regulating cytokines [7, 8].

Inflammation is a risk factor for mild cognitive impairment and AD [9, 10]. Epidemiological studies have found that elevated levels of systemic inflammation were associated with cognitive decline [11]. It seemed that both gut microbiota and cytokines can affect the development of dementia. We assumed that cytokines may be mediating factors in the pathway from gut microbiota to dementia.

Although randomized controlled trials could help establish causal relationships between gut microbiota or cytokines and dementia, they are difficult to perform in humans due to the limitation of objective conditions, such as the screening of strains and lowering the levels of cytokines. As a result, most of the current research conclusions are based on observations of the composition and changes in the gut microbiota in dementia patients' feces [6, 12], or indirect interventions such as probiotic supplementation [13], and aspirin or other non-steroidal anti-inflammatory drugs [14].

Genome-wide association studies (GWAS) have tested millions of genetic variants in many individual genomes to identify genotype–phenotype associations and have revolutionized the field of complex disease genetics in the past decade [15].

Mendelian randomization (MR) is a genetic epidemiological method. In MR, genetic variants are used as instrumental variables (IVs) for assessing the causal effect between exposure and outcome [16–19]. Genetic variants have been determined at the time of conception and therefore MR is less susceptible to environmental confounding factors and reverse causality compared with observational studies [16, 17]. A one sample MR analysis requires the exposure and outcome from the same individual whereas a two-sample MR analysis requires them from different GWAS summary databases. The two-sample MR method is greater statistical power to obtain the causal effects between "the exposure factors" and "the outcome" by taking advantage of published summary estimates from large scale different GWAS [20, 21]. Large-scale summary statistics were available to analyze the relationships between gut microbiota or cytokines and dementia, which improved the statistical power of two-sample MR analysis.

In this study, we conducted a comprehensive MR analysis to explore the causal effects between the gut microbiome, cytokines, and multiple dementia types (including AD, FTD, DLB, VD, and PDD). Then we explored whether cytokines as mediators in the pathway from gut microbiota to dementia. In addition, through reverse causality analysis, we can also examine whether genetic predisposition to dementia risk affects gut microbiota and cytokines.

## Methods

### Study design

**Fig. 1:**
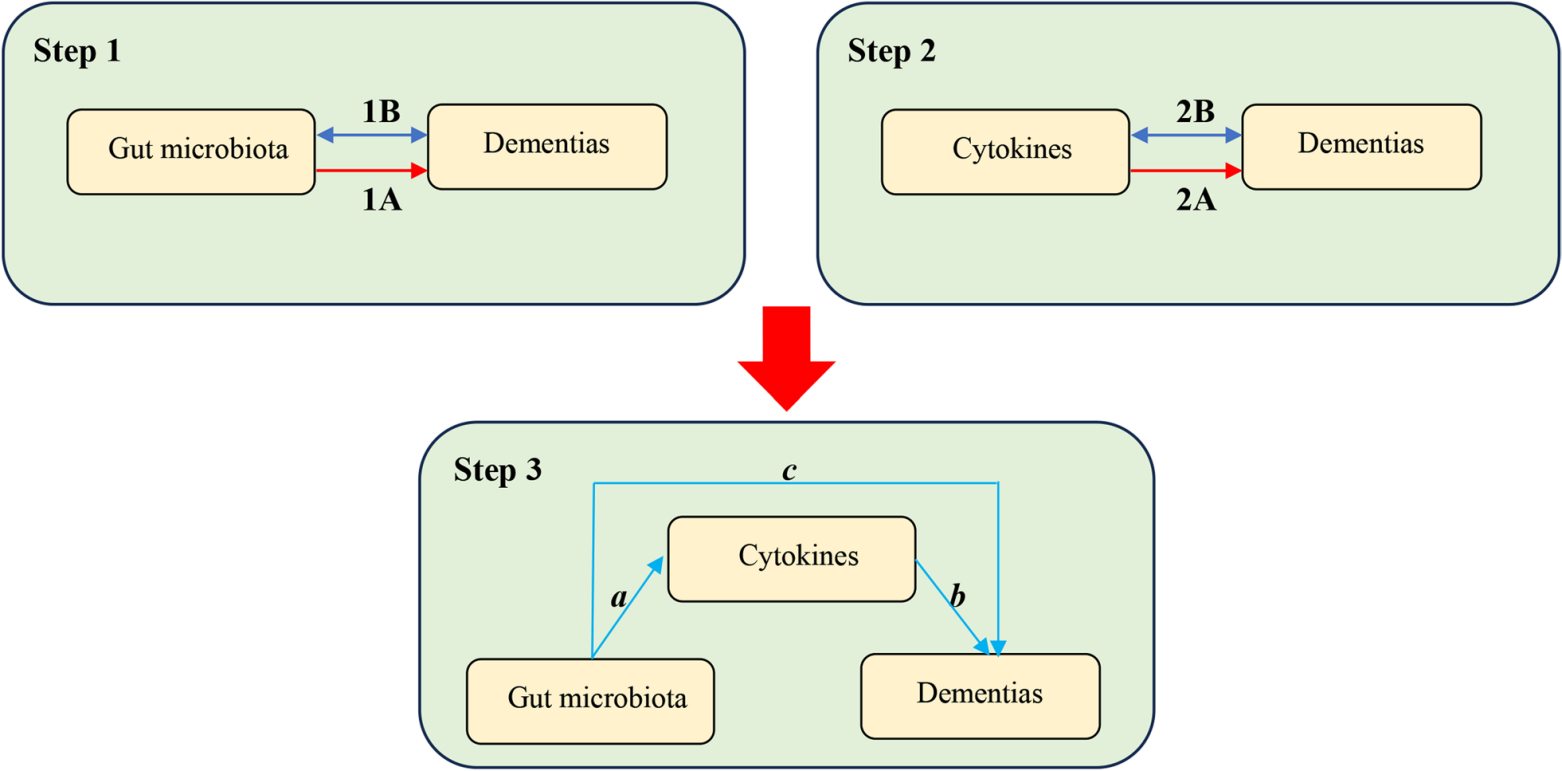
Study overview. Step 1A represents the causal effects of gut microbiota on dementia. Step 1B represents the bi‑directional causal effects between gut microbiota and dementia. Step 2A represents the causal effects of cytokines on dementia. Step 2B represents the bi‑directional causal effects between cytokines and dementia. Step 3 represents the mediating analysis of cytokines in the pathway from the gut microbiota to dementia: path *c* was the total effect of gut microbiota on dementia; path *b* was the causal effect of cytokines on dementia; path *a* was the causal effect of gut microbiota on cytokines

This study has three main components as outlined in Fig. 1: analysis of causal effects of 211 gut microbiota on five dementias (step 1A); analysis of causal effects of 41 cytokines on five dementias (step 2A); and mediation analysis of cytokines in the pathway from gut microbiota to dementias (step 3). We defined single-nucleotide polymorphisms (SNPs) as IVs. Mendelian randomization is based on three core assumptions: (1) the IVs are closely associated with the exposure factors; (2) IVs are not associated with confounding factors; (3) IVs do not affect the outcome directly, and it can only affect outcome via the exposure [22].

### Data source

The genetic data for the gut microbiome came from the latest GWAS summary data, in which the MiBioGen consortium curated and analyzed genome-wide genotypes and 16S fecal microbiome data from 18,340 individuals (24 cohorts) [23]. The GWAS summary data included a total of 211 gut microbiota taxa (131 genera, 35 families, 20 orders, 16 classes, and 9 phyla). The genetic data for cytokines came from the previously GWAS (8,337 individuals), including 41 cytokines [24].

The GWAS summary data of AD, FTD, VD, and PDD were derived from the eighth version of the Finngen consortium (https://r8.risteys.finngen.fi/). It was a prospective cohort study that patients were screened using International Classification of Diseases (ICD) diagnosis codes for four subtypes of dementia. We downloaded genetic data of four subtypes of dementia from the Finngen database, respectively. The GWAS summary data of DLB were derived from the study by chia et al. [25], and included in the IEU Open GWAS database (https://gwas.mrcieu.ac.uk/). Participants were recruited across 44 institutions/consortia and were diagnosed according to established consensus criteria. Detailed information is provided in Additional file 2: Table S1.

The present study is a secondary analysis of publicly available GWAS summary statistics. Ethical approval was granted for each of the original GWAS studies. In addition, no individual-level data was used in this study. Therefore, no new ethical review board approval was required.

### Instrumental variables selection

First, we selected the SNPs with significant associations for gut microbiota (*P* < 1 × 10^–5^). To maximize the number of available instruments for each cytokine, we selected the SNPs with a *P*-value of 5 × 10^–6^ as the threshold. Then we excluded the SNPs with linkage disequilibrium (LD) in the analysis. The LD of chosen SNPs strongly related to gut microbiota should meet the condition that r^2^ < 0.001 and distance > 10,000 kb [26]. An important step in MR analysis is to ensure that the effects of SNPs on exposure correspond to the same allele as the effects on outcome. After matching the outcome, we removed palindromic SNPs. (A palindromic SNP is an SNP with the A/T or G/C allele.)

We extracted the relevant information: chromosome, effect allele (EA), other allele (OA), effect allele frequency (EAF), effect sizes (β), standard error (SE), and P-value. Last, we calculated the explained variance (R^2^) and F-statistic parameters to determine whether the identified IVs were strongly associated with exposure. Generally, SNPs with F-statistic parameters < 10 are considered weak instruments [27]. In this study, R^2^ = 2 × EAF × (1-EAF) × β^2^ / (2 × EAF × (1-EAF) × β^2^ + 2 × EAF × (1-EAF) × N × SE^2^), where N is the sample size of the GWAS for FI, and F = R^2^ × (N-2)/(1-R^2^) [28].

### MR analysis

#### Primary analysis

To estimate the causal effects of gut microbiota and cytokines on dementia, we performed two-sample MR analysis, respectively (step 1A and step 2A in Fig. 1). The inverse variance weighted (IVW) approach was as the essential analysis method and the Wald ratios test for features containing only one IV [29]. MR results were expressed as odds ratios (ORs) with the corresponding 95% confidence intervals (CI). The results were statistically significant when P-value of IVW were less than 0.05 and the direction of IVW and MR-Egger were consistent. A two-sided P-value that passed the Bonferroni correction P-value was defined as statistically significant, which is 0.0012 (0.05/41) for cytokines. A P < 0.05, but above the Bonferroni-corrected threshold, was considered as suggestive for association.

#### Mediation analysis

By the two-sample analysis (step 1A and step 2A in Fig. 1), the gut microbiota and cytokines with significant causal effects on dementias were included in the mediation analysis. We explored whether gut microbiota had a causal effect on cytokines (step 3, path *a*, in Fig. 1), and if so, we would perform multiple MR analysis to explore whether cytokines were the mediation factors in the pathway from gut microbiota to dementia.

#### Bi‑directional causality analysis

To evaluate bi-directional causation effects between gut microbiota, cytokines, and dementias, we used dementias as “exposure” and gut microbiota or cytokines associated with dementias as “outcome” (step 1B and step 2B in Fig. 1). We selected the SNPs significantly associated with dementia (*P* < 5 × 10^–8^) as IVs.

### Sensitivity analysis

We performed Cochran's Q test to evaluate the heterogeneity of each SNP [30] and generated scatter plots of SNP–exposure associations and SNP–outcome associations to visualize MR results. Leave-one-out analysis was performed to evaluate if each SNP could affect the results (by excluding each SNP at a time sequentially and an IVW method was performed on the remaining SNPs to assess the potential influence of a particular variant on the estimates) [31]. In addition, we used MR-PRESSO and MR-Egger regression to test the potential horizontal pleiotropy effect. MR-PRESSO was used to detect significant outliers and to correct the horizontal plural effect by removing outliers [32].

All analyses were carried out using R (v4.2.1) statistical software. MR analysis was performed using the R-based package “TwoSampleMR”. The “MR_PRESSO” package was used for multiplicity tests [33].

## Results

### Instrumental variable selection

Initially, we identified 224, 478, 1667, 280, and 125 SNPs associated with 210 gut microbiotas at the class, family, genus, order, and phylum levels, respectively, at a level of *P* < 1 × 10^–5^ (one gut microbiota was excluded due to no eligible SNPs). These 2774 SNPs were selected as IVs for the 210 gut microbiota taxa (Additional file 3: Table S2). Then, we identified 451 SNPs associated with 41 cytokines at a level of *P* < 5 × 10^–6^ (Additional file 4: Table S3).

### Causal effects of gut microbiota and cytokines on multiple dementia types

#### AD

**Fig. 2:**
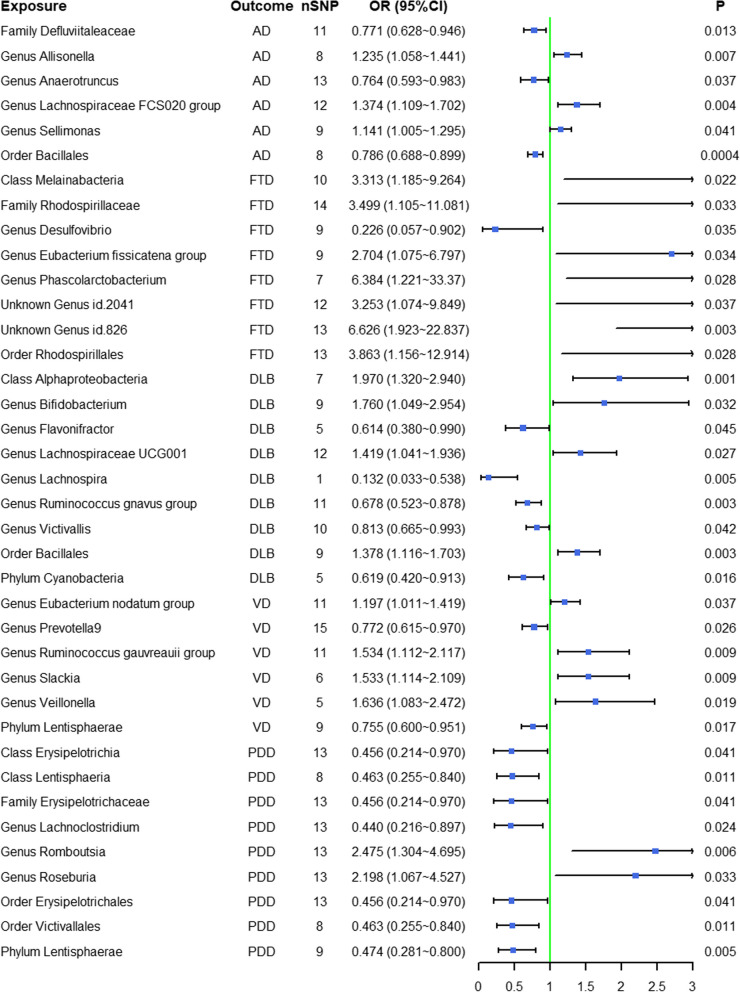
Mendelian randomization results of causal effects between gut microbiotas and five subtypes of dementia

A total of six gut microbiotas (including one family, four genera, and one order) were associated with AD (Additional file 5: Table S4, Fig. 2). Detailed 61 SNPs information for six gut microbiotas is shown in Additional file 6: Table S5.

As shown in Fig. 2, MR analysis suggested that genetic prediction of three gut microbiotas (genus *Allisonella*, genus *Lachnospiraceae FCS020 group*, and genus *Sellimonas*) was associated with an increased risk of AD. The genus *Allisonella* (OR = 1.235, 95%CI = 1.058 ~ 1.441, *P* = 0.007), genus *Lachnospiraceae FCS020 group* (OR = 1.374, 95%CI = 1.109 ~ 1.702, *P* = 0.004) significantly increased the risk of AD.

Genetic prediction of three gut microbiotas (family *Defluviitaleaceae*, genus *Anaerotruncus*, and order *Bacillales*) was associated with a decreased risk of AD. The family *Defluviitaleaceae* (OR = 0.771, 95%CI = 0.628 ~ 0.946, *P* = 0.013), and order *Bacillales* (OR = 0.786, 95%CI = 0.688 ~ 0.899, *P* < 0.001) significantly decreased the risk of AD.

**Fig. 3:**
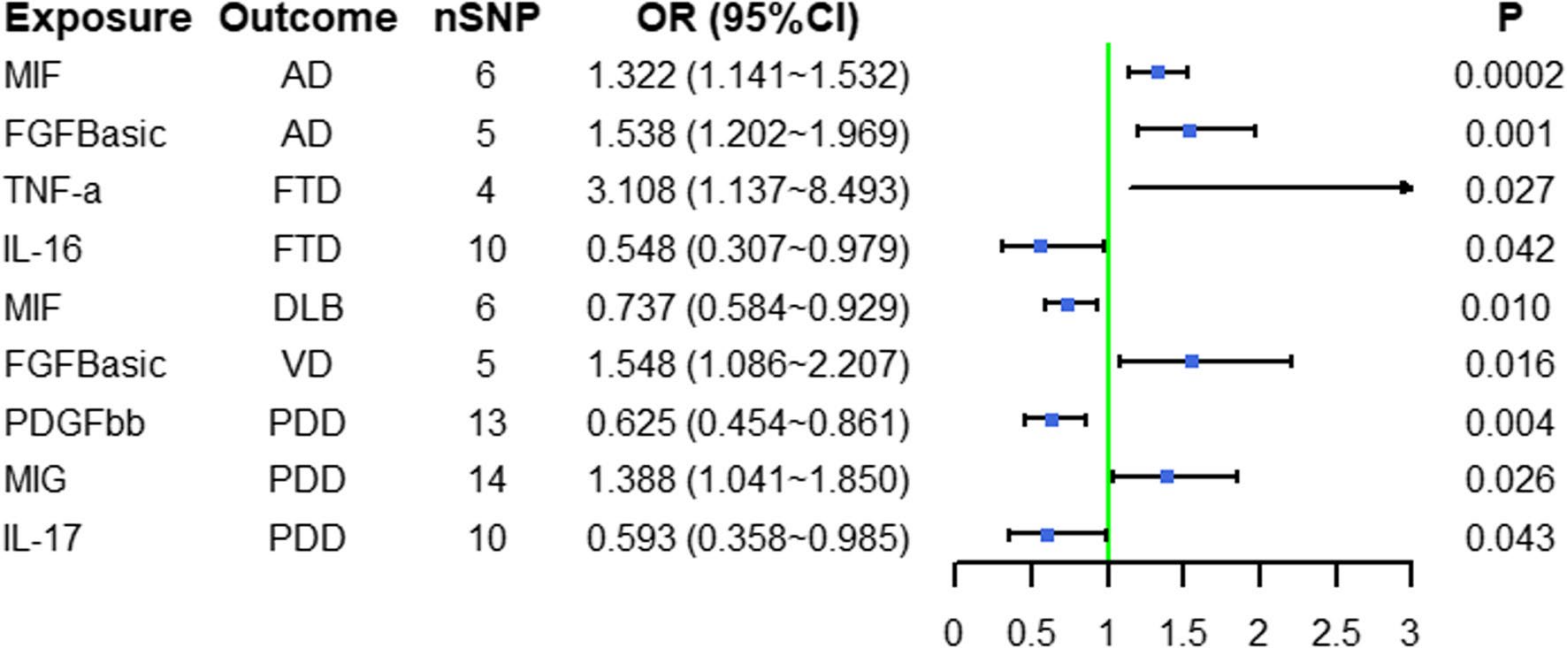
Mendelian randomization results of causal effects between cytokines and five subtypes of dementia

As shown in Fig. 3, macrophage migration inhibitory factor (MIF) (OR = 1.322, 95%CI = 1.141 ~ 1.532, *P* < 0.001) and basic fibroblast growth factor (FGFBasic) (OR = 1.538, 95%CI = 1.202 ~ 1.969, *P* = 0.001) significantly increased the incidence of AD (Additional file 7: Table S6).

#### FTD

A total of eight gut microbiota (including one class, one family, five genera, and one order) were associated with FTD (Additional file 5: Table S4, Fig. 2). Detailed 87 SNPs information for the eight gut microbiotas is shown in Additional file 6: Table S5.

Figure 2 shows that genetic prediction of seven gut microbiotas (class *Melainabacteria*, family *Rhodospirillaceae*, genus *Eubacterium fissicatena group*, genus *Phascolarctobacterium*, unknown genus id.2041, unknown genus id.826, and order *Rhodospirillales*) was associated with an increased risk of FTD. The class *Melainabacteria* (OR = 3.313, 95%CI = 1.185 ~ 9.264, *P* = 0.022), unknown genus id.826 (OR = 6.626, 95%CI = 1.923 ~ 22.837, *P* = 0.003), and order *Rhodospirillales* (OR = 3.863, 95%CI = 1.156 ~ 12.914, *P* = 0.028) significantly increased the risk of FTD.

The genus *Desulfovibrio* (OR = 0.226, 95%CI = 0.057 ~ 0.902, *P* = 0.035) was associated with a decreased risk of FTD.

Figure 3 shows that tumor necrosis factor-alpha (TNF-α) and interleukin-16 (IL-16) were associated with FTD (Additional file 7: Table S6). TNF-α (OR = 3.108, 95%CI = 1.137 ~ 8.493, *P* = 0.027) significantly increased the incidence of FTD.

#### DLB

A total of nine gut microbiotas (including one class, six genera, one order, and one phylum) were associated with DLB (Additional file 5: Table S4, Fig. 2). Detailed 69 SNPs information for the nine gut microbiotas is shown in Additional file 6: Table S5.

As shown in Fig. 2, genetic prediction of four gut microbiotas (class *Alphaproteobacteria*, genus *Bifidobacterium*, genus *Lachnospiraceae UCG001*, and order *Bacillales*) was associated with an increased risk of DLB. The class *Alphaproteobacteria* (OR = 1.970, 95%CI = 1.320 ~ 2.940, *P* = 0.001) and order *Bacillales* (OR = 1.378, 95%CI = 1.116 ~ 1.703, *P* = 0.003) significantly increased the risk of DLB.

Genetic prediction of five gut microbiotas (genus *Flavonifractor*, genus *Lachnospira*, genus *Ruminococcus gnavus group*, genus *Victivallis*, and phylum *Cyanobacteria*) was associated with a decreased risk of DLB. The genus *Lachnospira* (OR = 0.132, 95%CI = 0.033 ~ 0.538, *P* = 0.005), genus *Ruminococcus gnavus group* (OR = 0.678, 95%CI = 0.523 ~ 0.878, *P* = 0.003), and phylum *Cyanobacteria* (OR = 0.619, 95%CI = 0.42 ~ 0.913, *P* = 0.016) significantly decreased the risk of DLB.

As shown in Fig. 3, MIF (OR = 0.737, 95%CI = 0.584 ~ 0.929, *P* = 0.010) seemed to be a protective factor for DLB (Additional file 7: Table S6).

#### VD

A total of six gut microbiotas (including five genera and one phylum) were associated with VD (Additional file 5: Table S4, Fig. 2). Detailed 57 SNPs information for the six gut microbiotas is shown in Additional file 6: Table S5.

As shown in Fig. 2, genetic prediction of four gut microbiotas (genus *Eubacterium nodatum group*, genus *Ruminococcus gauvreauii group*, genus *Slackia*, and genus *Veillonella*) was associated with an increased risk of VD. The genus *Ruminococcus gauvreauii group* (OR = 1.534, 95%CI = 1.112 ~ 2.117, *P* = 0.009), genus *Slackia* (OR = 1.533, 95%CI = 1.114 ~ 2.109, *P* = 0.009), and genus *Veillonella* (OR = 1.636, 95%CI = 1.083 ~ 2.472, *P* = 0.019) significantly increased risk of VD.

Genetic prediction of two gut microbiotas was associated with a decreased risk of VD. The genus *Prevotella9* (OR = 0.772, 95%CI = 0.615 ~ 0.970, *P* = 0.026) and phylum *Lentisphaerae* (OR = 0.755, 95%CI = 0.600 ~ 0.951, *P* = 0.017) decreased the risk of VD.

FGFBasic (OR = 1.548, 95%CI = 1.086 ~ 2.207, *P* = 0.016) was associated with VD (Fig. 3, Additional file 7: Table S6).

#### PDD

A total of nine gut microbiota (including two classes, one family, three genera, two orders and one phylum) were associated with PDD (Additional file 5: Table S4, Fig. 2). Detailed 103 SNPs information for nine gut microbiotas is shown in Additional file 6: Table S5.

As shown in Fig. 2, genetic prediction of two gut microbiotas was associated with an increased risk of PDD. The genus *Romboutsia* (OR = 2.475, 95%CI = 1.304 ~ 4.695, *P* = 0.006) and genus *Roseburia* (OR = 2.198, 95%CI = 1.067 ~ 4.527, *P* = 0.033) increased the risk of PDD.

Genetic prediction of seven gut microbiotas was associated with a decreased risk of PDD. Notably, the class *Erysipelotrichia*, family *Erysipelotrichaceae*, and order *Erysipelotrichales* were the same gut microbiota. The class *Lentisphaeria* (OR = 0.463, 95%CI = 0.255 ~ 0.840, *P* = 0.011), genus *Lachnoclostridium* (OR = 0.440, 95%CI = 0.216 ~ 0.897, *P* = 0.024), order *Victivallales* (OR = 0.463, 95%CI = 0.255 ~ 0.840, *P* = 0.011), and phylum *Lentisphaerae* (OR = 0.474,95%CI = 0.281 ~ 0.800, *P* = 0.005) significantly decreased the risk of PDD.

A total of three cytokines were associated with PDD, including platelet-derived growth factor BB (PDGFbb), monokine induced by interferon-gamma (MIG), and interleukin-17 (IL-17) (Fig. 3, Additional file 7: Table S6). PDGFbb (OR = 0.625,95%CI = 0.454 ~ 0.861, *P* = 0.004) had a protective causal effect on PD. MIG (OR = 1.388,95%CI = 1.041 ~ 1.850, *P* = 0.026) was a risk factor for PDD.

### Sensitivity analyses

According to MR-Egger regression intercept approach, genetic pleiotropy did not bias the results, and MR-PRESSO analysis proved that there was no horizontal pleiotropy in the MR study (*P* > 0.05, Additional file 8: Table S7). The Cochran’s Q tests showed no significant heterogeneity (*P* > 0.05, Additional file 8: Table S7).

The results of “leave-one-out” analysis proved that MR analysis turned out to be reliable. (The null line is not within the total confidence interval of the SNPs, Additional file 1: Figure S1-5.) The scatter plots showed the overall effect of gut microbiota on dementia (Additional file 1: Figure S6-10). In addition, the forest plots indicated the causal associations between gut microbiota and dementia (Additional file 1: Figure S11-15).

### Bi‑directional causal effects of dementias on gut microbiota and cytokines

As shown in Additional file 9: Table S8, there was no reverse effect between gut microbiota, cytokines, and AD. After matching FTD and gut microbiota or cytokines, no SNP can be used as IV. DLB had causal effects on genus *Lachnospira* (OR = 0.833, 95%CI = 0.755 ~ 0.919, *P* < 0.001) and order *Bacillales* (OR = 1.075, 95%CI = 1.005 ~ 1.150, *P* = 0.034). VD had causal effects on genus *Veillonella* (OR = 1.071, 95%CI = 1.003 ~ 1.143, *P* = 0.040) and FGFBasic (OR = 1.085, 95%CI = 1.016 ~ 1.157, *P* = 0.014). PDD had a causal effect on *Erysipelotrichia* (OR = 1.070, 95%CI = 1.005 ~ 1.139, *P* = 0.034).

### Mediation analysis

In this study, gut microbiota and cytokines all had causal effects on dementias. It seemed that cytokines played a mediating effect in the pathway from gut microbiota and dementia. One of the requirements for mediating effect is that gut microbiota was significantly associated with cytokines. However, our results revealed that there were no causal effects between gut microbiotas associated with dementias and cytokines associated with dementias (step 3*a* in Fig. 1; Additional file 10: Table S9), which indicated that cytokines did not act as a mediator in the pathway from gut microbiotas and dementias.

## Discussion

The gut microbiome assists in many daily functions of the brain, including regulating the activation state of the hypothalamic–pituitary–adrenal (HPA) axis and activating the vagus and adrenergic nerves; in addition, intestinal bacteria themselves can synthesize and release many neurotransmitters and neuromodulators, or stimulate intestinal endocrine cells to synthesize and release neuropeptides [3].

The maladjustment of the brain–intestine–microbiome axis may lead to the dysfunction of the intestinal epithelial barrier, which in turn promotes the invasion of neuroactive substances, including neurotropic viruses and so on [34]. The gut microbiome may also play a role in metabolic diseases, such as insulin resistance and fatty liver disease [35]. Studies have shown that cholesterol metabolism is related to the mechanism of dementia development [36].

Studies have indicated that gut microbiota might affect dementia development. Naoki performed a cross-sectional study revealing that the number of Bacteroides (enterotype I) was lower in demented than non-demented patients [6]. However, due to the different types of dementia and the complexity of the gut microbiota, it was difficult to adequately summarize the gut microbiota affecting dementia through observational studies.

In this study, we used an MR study to explore the potential causal effects between gut microbiota and dementia. We analyzed the relationships between 210 common gut microbiota abundance and five types of dementia (AD, FTD, DLB, VD, PDD). The results showed that some gut microbiotas were risk factors, and some were protective factors for each dementia subtype.

High abundance of *Allisonella*, *Lachnospiraceae FCS020 group*, and *Sellimonas* could increase the risk of AD. *Allisonella* was associated with high level of inflammation [37], which provided a hypothesis on how *Allisonella* increased the risk of AD. A high abundance of *Defluviitaleaceae*, *Anaerotruncus*, and *Bacillale* could decrease the risk of AD.

Few studies explored the association between gut microbiota and FTD. In this study, *Melainabacteria*, *Rhodospirillaceae*, *Eubacterium fissicatena group*, *Phascolarctobacterium*, unknown genus id.2041, unknown genus id.826, and *Rhodospirillales* may be risk factors for FTD, and *Desulfovibrio* seemed to be a protective factor for FTD. Further studies were necessary considering there were only 103 cases of FTD.

In DLB, genus *Ruminococcus* may mitigate neuroinflammation in the substantia nigra by increasing secondary bile acids [38], which may explain why *Ruminococcus* decreased the risk of DLB. In addition, *Flavonifractor*, *Lachnospira*, *Victivallis*, and *Cyanobacteria* were also protective factors for DLB. *Alphaproteobacteria*, *Bifidobacterium*, *Lachnospiraceae UCG001*, and *Bacillales* may increase the incidence of DLB.

The evidence regarding whether the specific gut microbiota affected VD remained unclear. By MR analysis, we found *Eubacterium nodatum group*, *Ruminococcus gauvreauii group*, *Slackia*, and *Veillonella* may be the risk factors for VD; *Prevotella9* and *Lentisphaerae* may decrease the incidence of VD.

Xie et al. reported that lower levels of *Romboutsia* and *Roseburia* were related to depressive symptoms in PD patients [39]. It seemed that a high abundance of *Romboutsia* and *Roseburia* could improve the symptom of PD patients. Contrary to their conclusions, we found that *Romboutsia* and *Roseburia* were associated with an increased risk of PDD. In terms of protective factors for PDD, our findings were similar to those of recent studies that in PD patients, Erysipelotrichaceae were markedly lowered, which proved that a higher abundance of Erysipelotrichaceae decreased the risk of PDD [40].

This study determined whether gut microbes were "helpful" or "harmful" to dementia by their relative abundance expression. However, the exact mechanism by which the gut microbiota causes dementia has not been determined. We assumed that cytokines may be mediating factors between gut microbiota and dementias.

According to MR analysis, we found that MIF and FGFBasic significantly increased the risk of AD. MIF is a pro-inflammatory cytokine. Previous studies have shown that increased MIF level could be a potential AD biomarker [41]. However, it seemed to be a negative correlation between MIF and DLB. TNF-α was significantly associated with FTD, which may be related to central degeneration [42].

In addition, dementia itself may affect changes in gut microbiota and cytokines. Therefore, we explored the causal effects of five dementia subtypes on gut microbiota and cytokines. The results showed that DLB had bi-directional causal effects on *Lachnospira* and *Bacillales*; VD had a bi-directional causal effect on *Veillonella* and FGFBasic; PDD had a bi-directional causal effect on *Erysipelotrichia*.

This was the first study to conduct a large-scale MR analysis of the causal relationships between the gut microbiome, cytokines, and several dementia subtypes. Our study had some limitations. First, the cases of dementia subtypes, especially FTD and PDD, were insufficient. Second, our study only analyzed the European population. Third, the 41 cytokines were derived from the blood, not the cerebrospinal fluid. Last, though we explored the mediating effects of cytokines between the abundance of different gut microbiota and dementias, the mechanisms how gut microbiota affected the onset of dementias remained to be studied considering that cytokines did not act as a mediating factor.

## Conclusion

In this study, we comprehensively explored the causal effects between gut microbiota, cytokines, and dementias. There were 20 positive and 16 negative causal effects between genetic liability in the gut microbiota and dementias. There were five positive correlations and four negative causal effects between cytokines and dementias. In addition, we found four bi-directional causal effects between the gut microbiota and dementias, and one between cytokines and dementias. Cytokines seemed not to act as a mediating factor in the pathway from gut microbiota to dementias.

### Supplementary Information

**Additional file 1:** The plots of MR analysis results.

**Additional file 2: Table S1.** Overview of the source of dementia data.

**Additional file 3: Table S2.** 2774 SNPs for the 210 gut microbiota taxa.

**Additional file 4: Table S3.** 451 SNPs for the 41 cytokines.

**Additional file 5: Table S4.** The causal effects of gut microbiota on dementia. 

**Additional file 6: Table S5.** The characteristics of SNPs analyzing the causal effects of gut microbiota on dementia.

**Additional file 7: Table S6.** The causal effects of cytokines on dementia. 

**Additional file 8: Table S7.** Mendelian randomization Sensitivity analysis. 

**Additional file 9: Table S8.** The causal effects of dementia on gut microbiota and cytokines. 

**Additional file 10: Table S9.** Causal effects of gut microbiota associated with dementia on cytokines associated with dementia. 

## Data Availability

All data used in the present study were obtained from genome‑wide association study summary statistics which were publicly released by genetic consortia.

## Abbreviations

AD: Alzheimer’s disease
FTD: Frontotemporal dementia
DLB: Dementia with Lewy body
VD: Vascular dementia
PD: Parkinson’s disease
PDD: Parkinson’s disease dementia
GWAS: Genome-wide association studies
MR: Mendelian randomization
IVs: Instrumental variables
SNP: Single-nucleotide polymorphism
ICD: International Classification of Diseases
LD: Linkage disequilibrium
EA: Effect allele
OA: Other allele
EAF: Effect allele frequency
β: Effect sizes
SE: Standard error
IVW: Inverse variance weighted
OR: Odds ratio
CI: Confidence interval
