# Supplementary material for: Gut microbiota, circulating cytokines and dementia: a Mendelian randomization study
Source: J Neuroinflammation. 2024 Jan 4;21:2. doi: 10.1186/s12974-023-02999-0 (PMC10765696; doi:10.1186/s12974-023-02999-0)
Supplement: Supplementary file 1 — Additional file 1: The plots of MR analysis results. [file 12974_2023_2999_MOESM1_ESM.docx]

**Additional Figures**

**Figure S1.** MR leave-one-out sensitivity analysis for Gut microbiota on AD.

**Figure S2.** MR leave-one-out sensitivity analysis for Gut microbiota on FTD.

**Figure S3.** MR leave-one-out sensitivity analysis for Gut microbiota on DLB.

**Figure S4.** MR leave-one-out sensitivity analysis for Gut microbiota on VD.

**Figure S5.** MR leave-one-out sensitivity analysis for Gut microbiota on PDD.

**Figure S6.** Scatter plots for the effect of Gut microbiota on AD.

**Figure S7.** Scatter plots for the effect of Gut microbiota on FTD.

**Figure S8.** Scatter plots for the effect of Gut microbiota on DLB.

**Figure S9.** Scatter plots for the effect of Gut microbiota on VD.

**Figure S10.** Scatter plots for the effect of Gut microbiota on PDD.

**Figure S11.** Forest plots for the effect of Gut microbiota on AD.

**Figure S12.** Forest plots for the effect of Gut microbiota on FTD.

**Figure S13.** Forest plots for the effect of Gut microbiota on DLB.

**Figure S14.** Forest plots for the effect of Gut microbiota on VD.

**Figure S15.** Forest plots for the effect of Gut microbiota on PDD.

**Figure S1.** MR leave-one-out sensitivity analysis for Gut microbiota on AD.


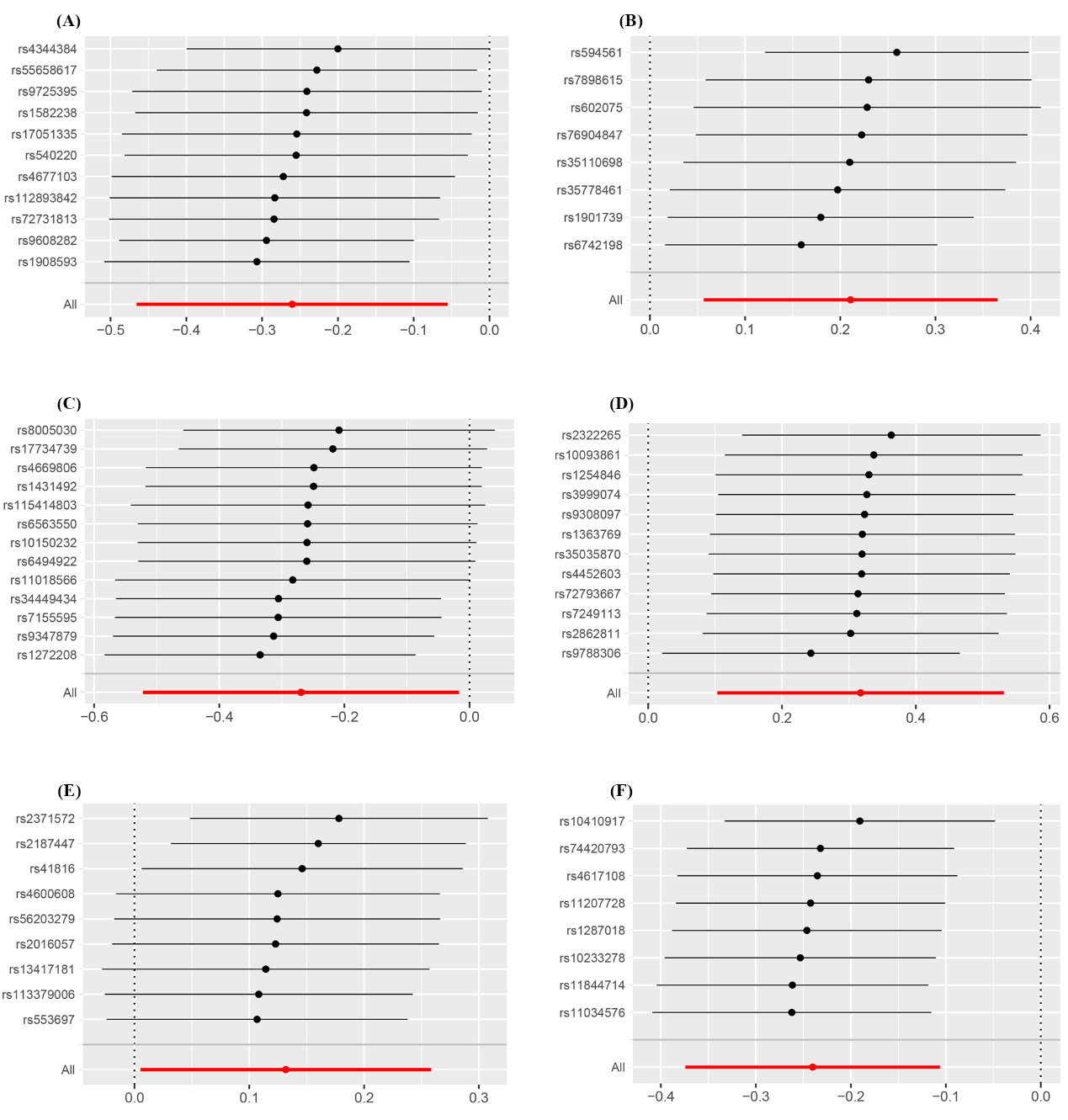


1. Analysis for "Family Defluviitaleaceae id.1924" on "AD"

(B) Analysis for "Genus Allisonella id.2174" on "AD"

(C) Analysis for "Genus Anaerotruncus id.2054" on "AD"

(D) Analysis for "Genus Lachnospiraceae FCS020 group id.11314" on "AD"

(E) Analysis for "Genus Sellimonas id.14369" on "AD"

(F) Analysis for "Order Bacillales id.1674" on "AD"

**Figure S2.** MR leave-one-out sensitivity analysis for Gut microbiota on FTD.


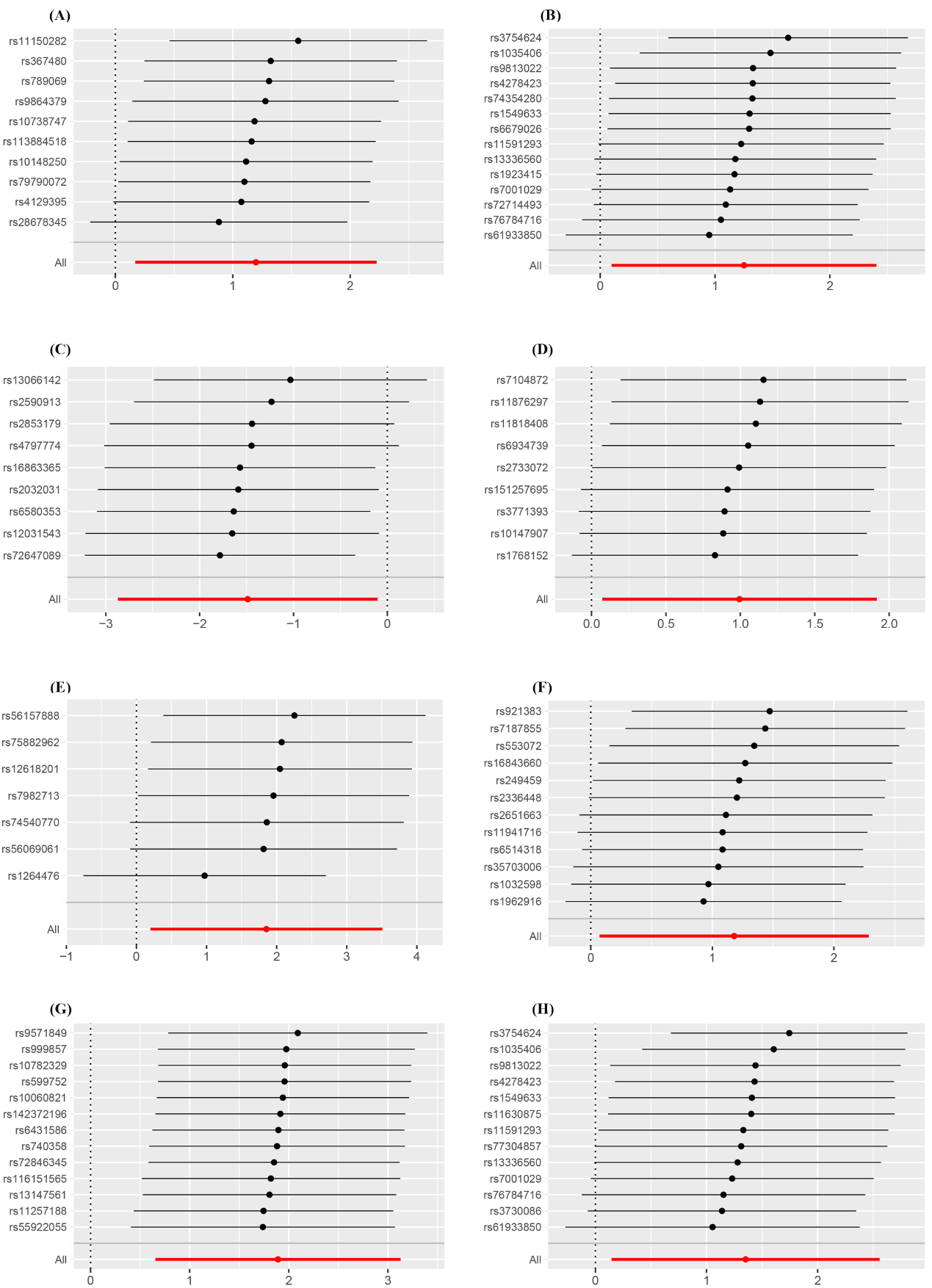


(A) Analysis for "Class Melainabacteria id.1589" on "FTD"

(B) Analysis for "Family Rhodospirillaceae id.2717" on "FTD"

(C) Analysis for "Genus Desulfovibrio id.3173" on "FTD"

(D) Analysis for "Genus Eubacterium fissicatena group id.14373" on "FTD"

(E) Analysis for "Genus Phascolarctobacterium id.2168" on "FTD"

(F) Analysis for "Unknown genus id.2041" on "FTD"

(G) Analysis for "Unknown genus id.826" on "FTD"

(H) Analysis for "Order Rhodospirillales id.2667" on "FTD"

**Figure S3.** MR leave-one-out sensitivity analysis for Gut microbiota on DLB.


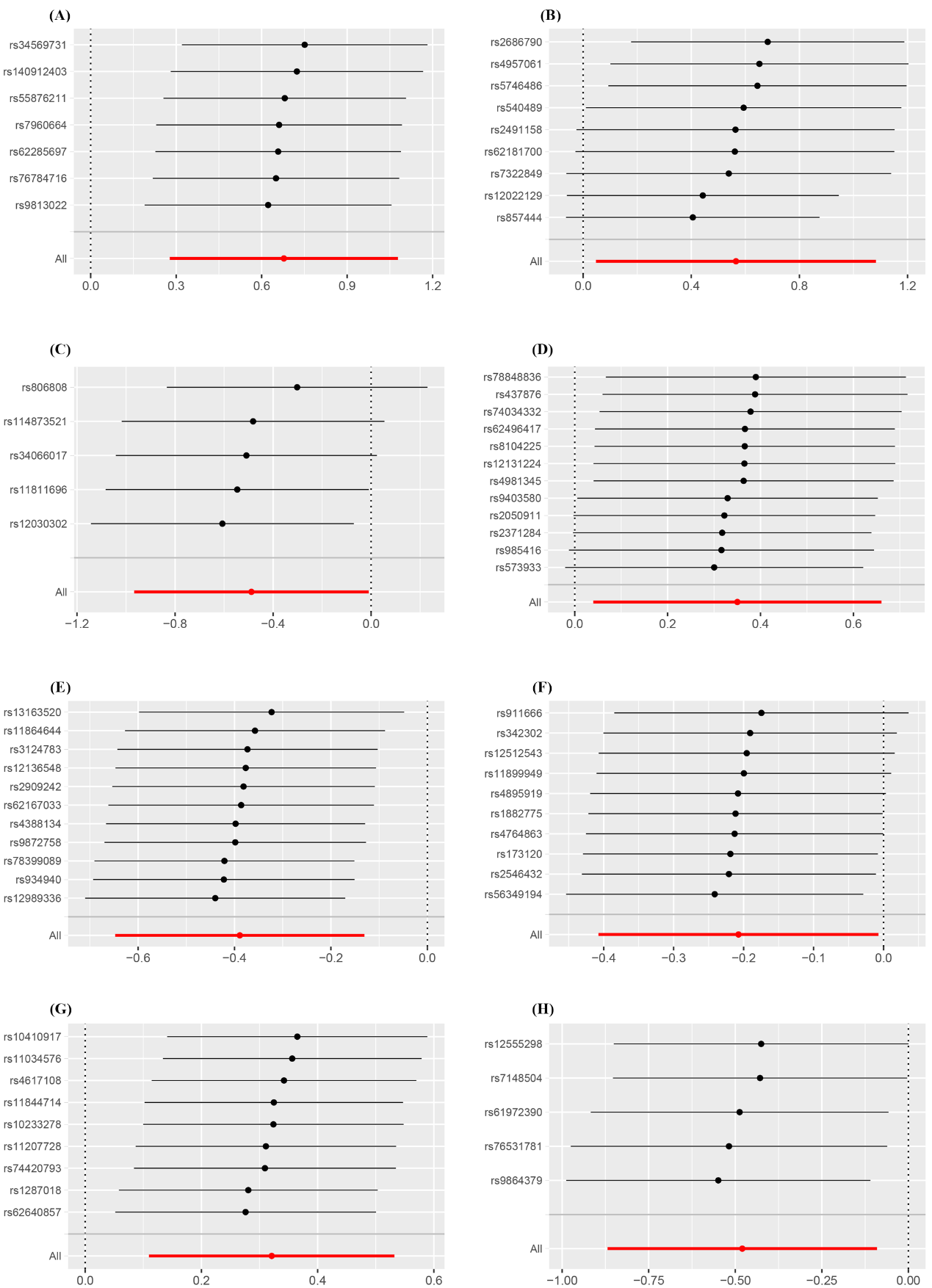


(A) Analysis for "Class Alphaproteobacteria id.2379" on "DLB"

(B) Analysis for "Genus Bifidobacterium id.436" on "DLB"

(C) Analysis for "Genus Flavonifractor id.2059" on "DLB"

(D) Analysis for "Genus Lachnospiraceae UCG001 id.11321" on "DLB"

(E) Analysis for "Genus Ruminococcus gnavus group id.14376" on "DLB"

(F) Analysis for "Genus Victivallis id.2256" on "DLB"

(G) Analysis for "Order Bacillales id.1674" on "DLB"

(H) Analysis for "Phylum Cyanobacteria id.1500" on "DLB"

**Figure S4.** MR leave-one-out sensitivity analysis for Gut microbiota on VD.


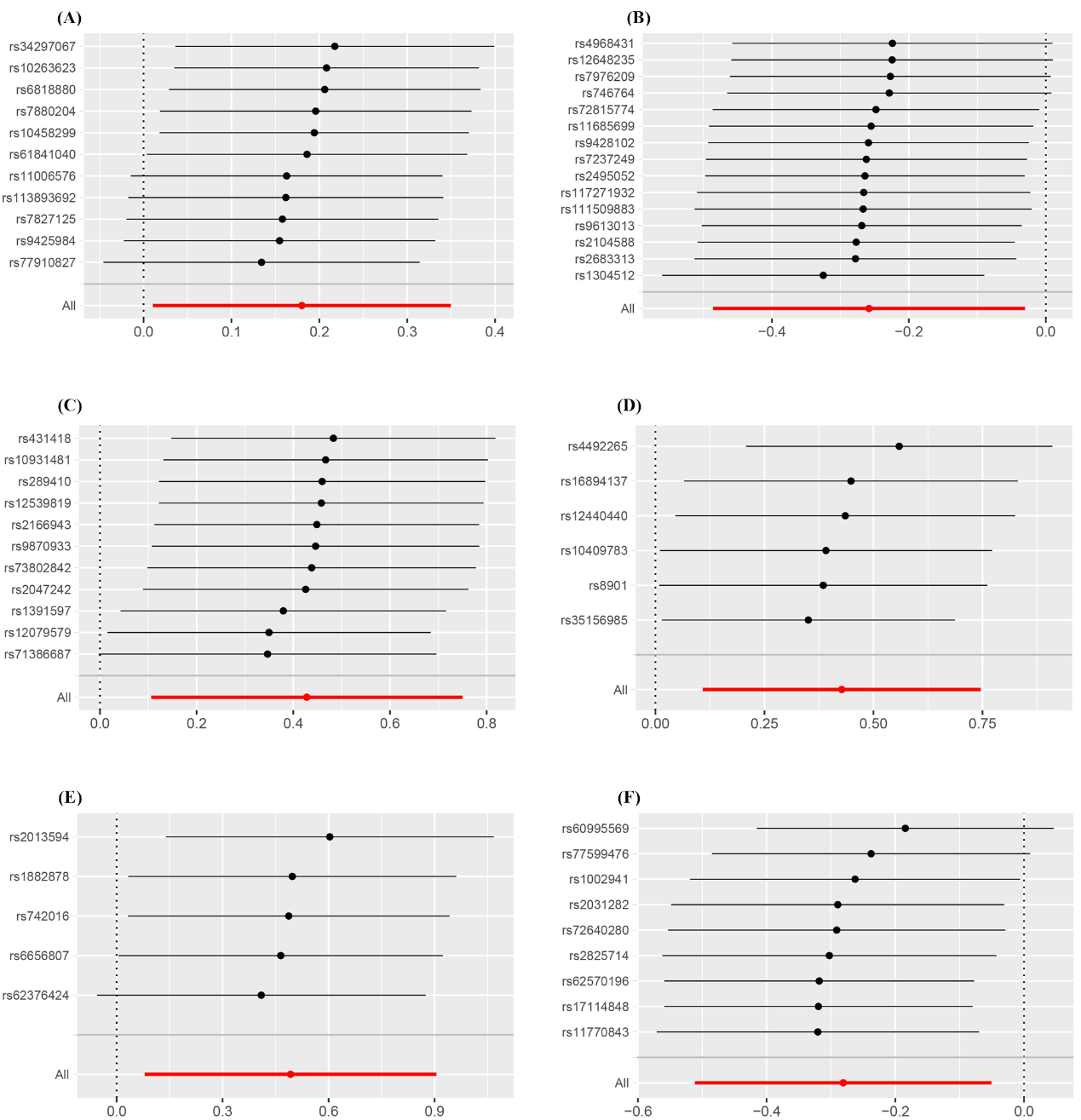


(A) Analysis for "Genus Eubacterium nodatum group id.11297" on "VD"

(B) Analysis for "Genus Prevotella9 id.11183" on "VD"

(C) Analysis for "Genus Ruminococcus gauvreauii group id.11342" on "VD"

(D) Analysis for "Genus Slackia id.825" on "VD"

(E) Analysis for "Genus Veillonella id.2198" on "VD"

(F) Analysis for "Phylum Lentisphaerae id.2238" on "VD"

**Figure S5.** MR leave-one-out sensitivity analysis for Gut microbiota on PDD.


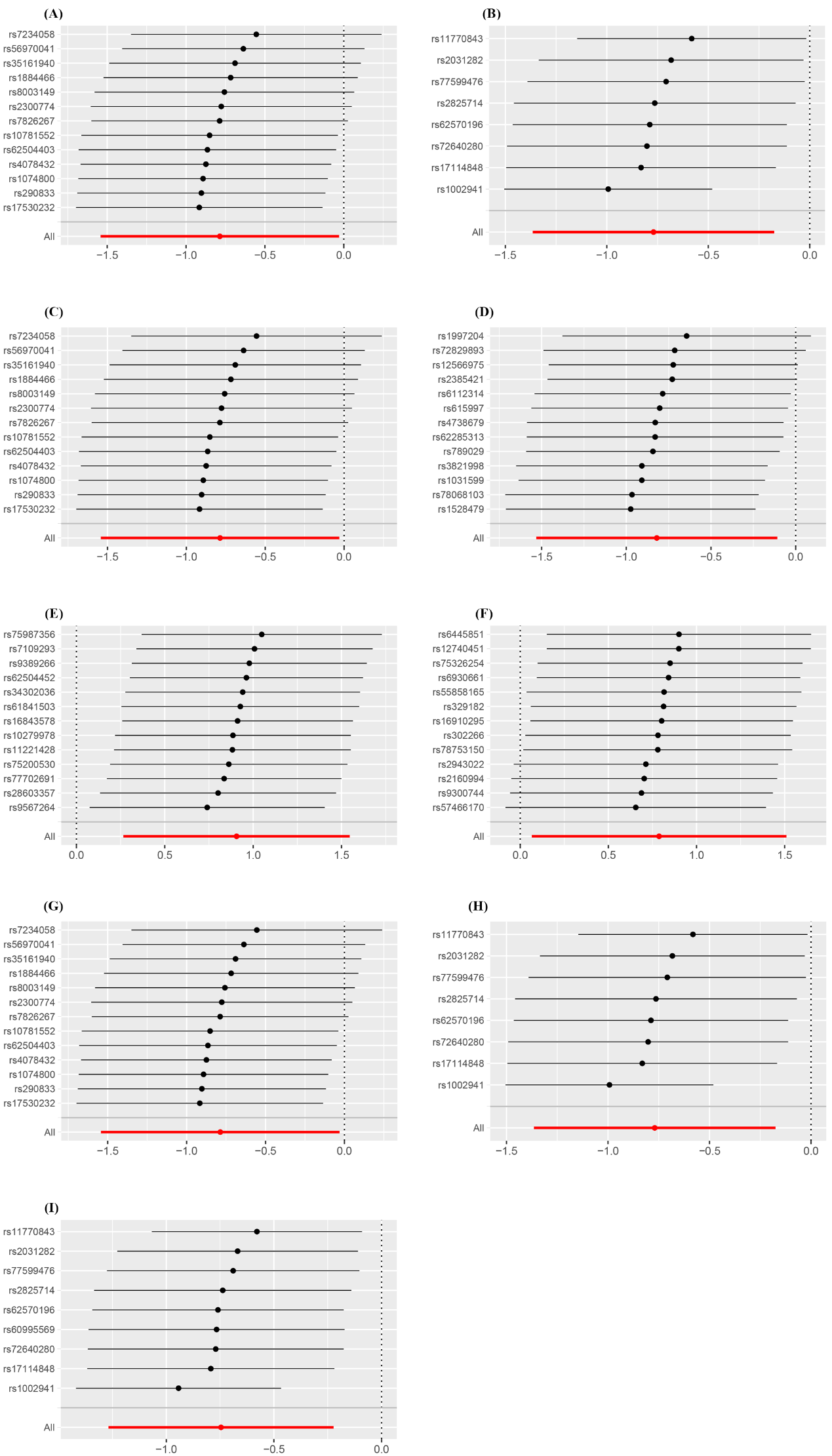


(A) Analysis for "Class Erysipelotrichia id.2147" on "PDD"

(B) Analysis for "Class Lentisphaeria id.2250" on "PDD"

(C) Analysis for "Family Erysipelotrichaceae id.2149" on "PDD"

(D) Analysis for "Genus Lachnoclostridium id.11308" on "PDD"

(E) Analysis for "Genus Romboutsia id.11347" on "PDD"

(F) Analysis for "Genus Roseburia id.2012" on "PDD"

(G) Analysis for "Order Erysipelotrichales id.2148" on "PDD"

(H) Analysis for "Order Victivallales id.2254" on "PDD"

(I) Analysis for "Phylum Lentisphaerae id.2238" on "PDD"

**Figure S6.** Scatter plots for the effect of Gut microbiota on AD.


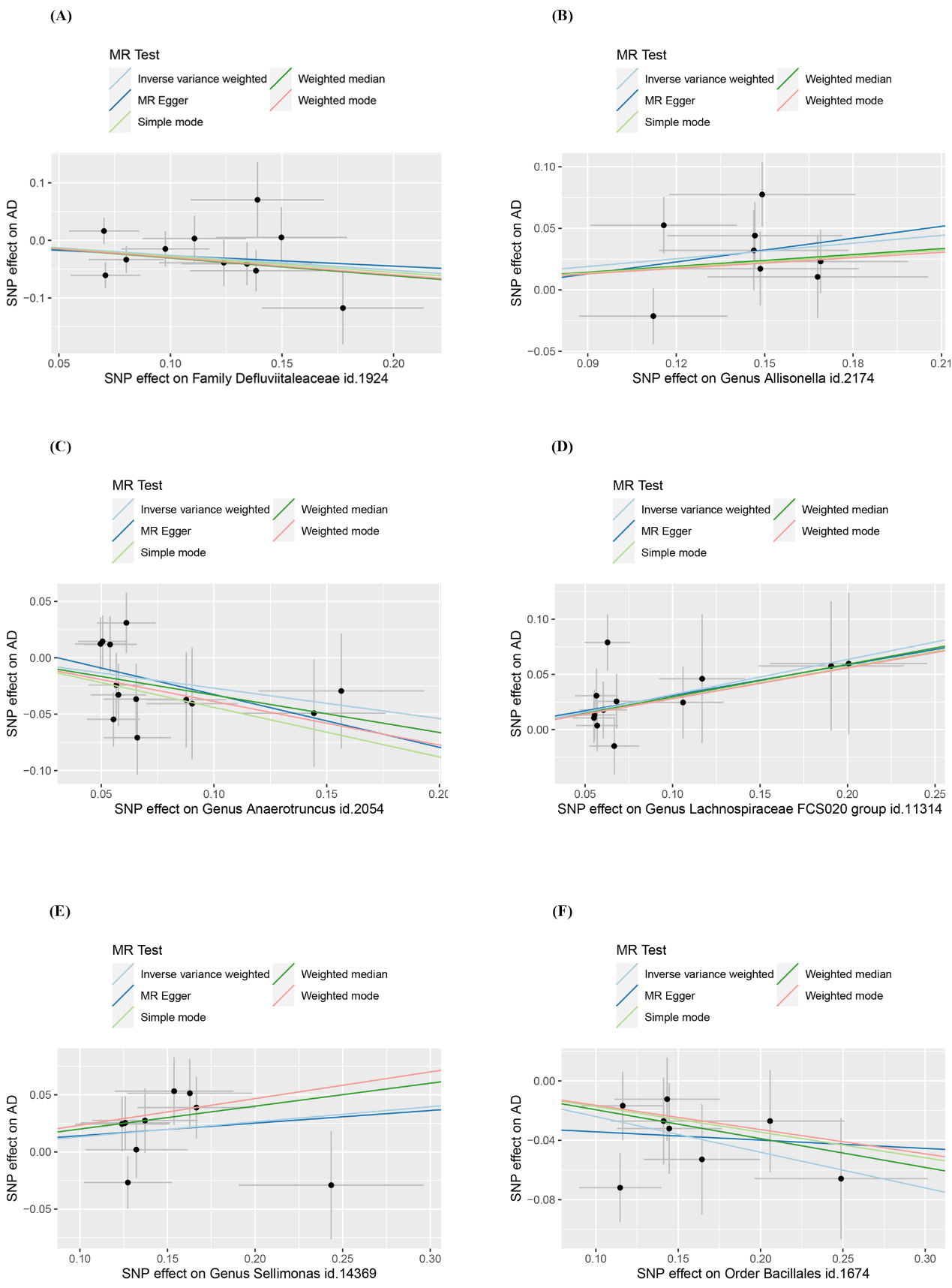


**Figure S7.** Scatter plots for the effect of Gut microbiota on FTD.


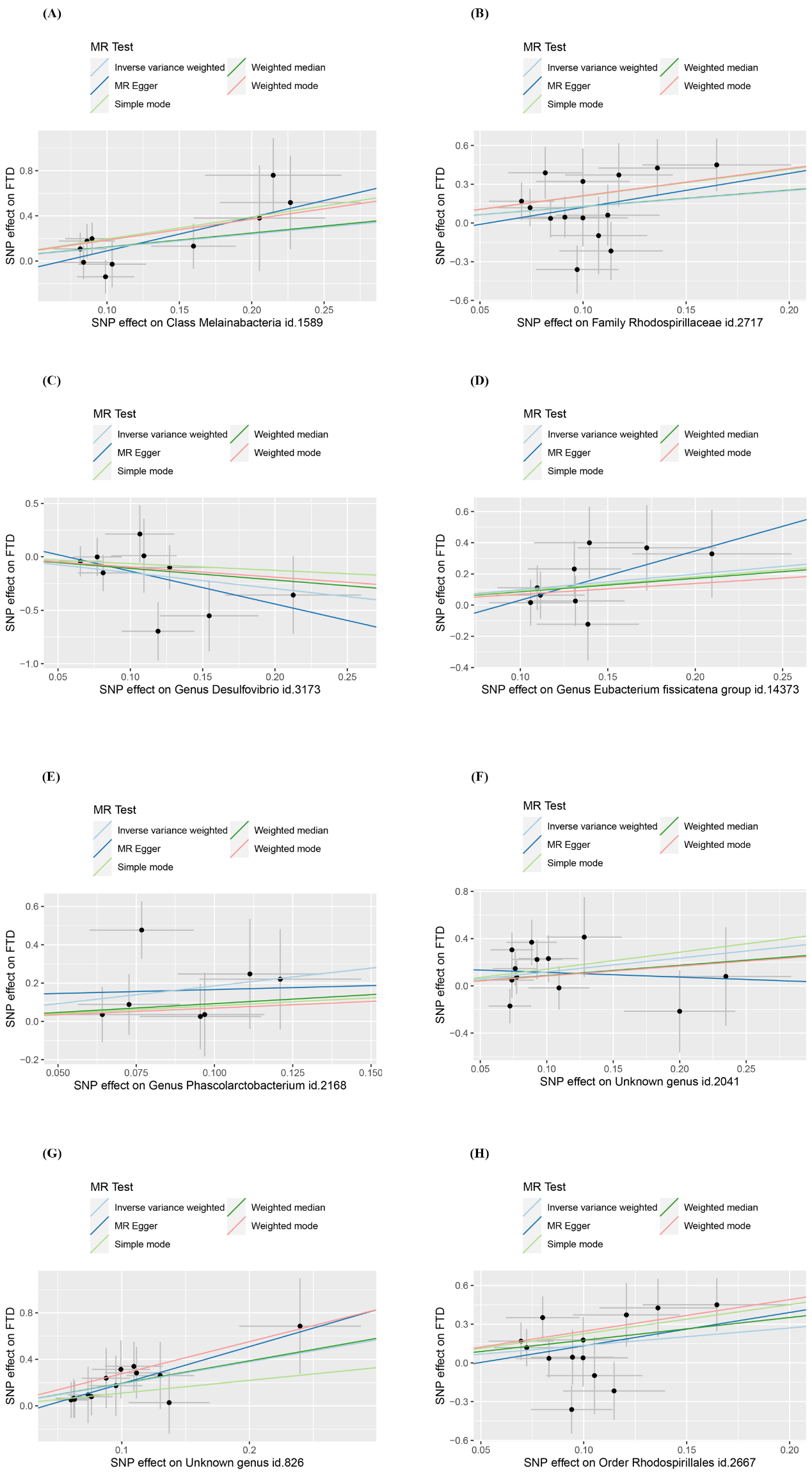


**Figure S8.** Scatter plots for the effect of Gut microbiota on DLB.


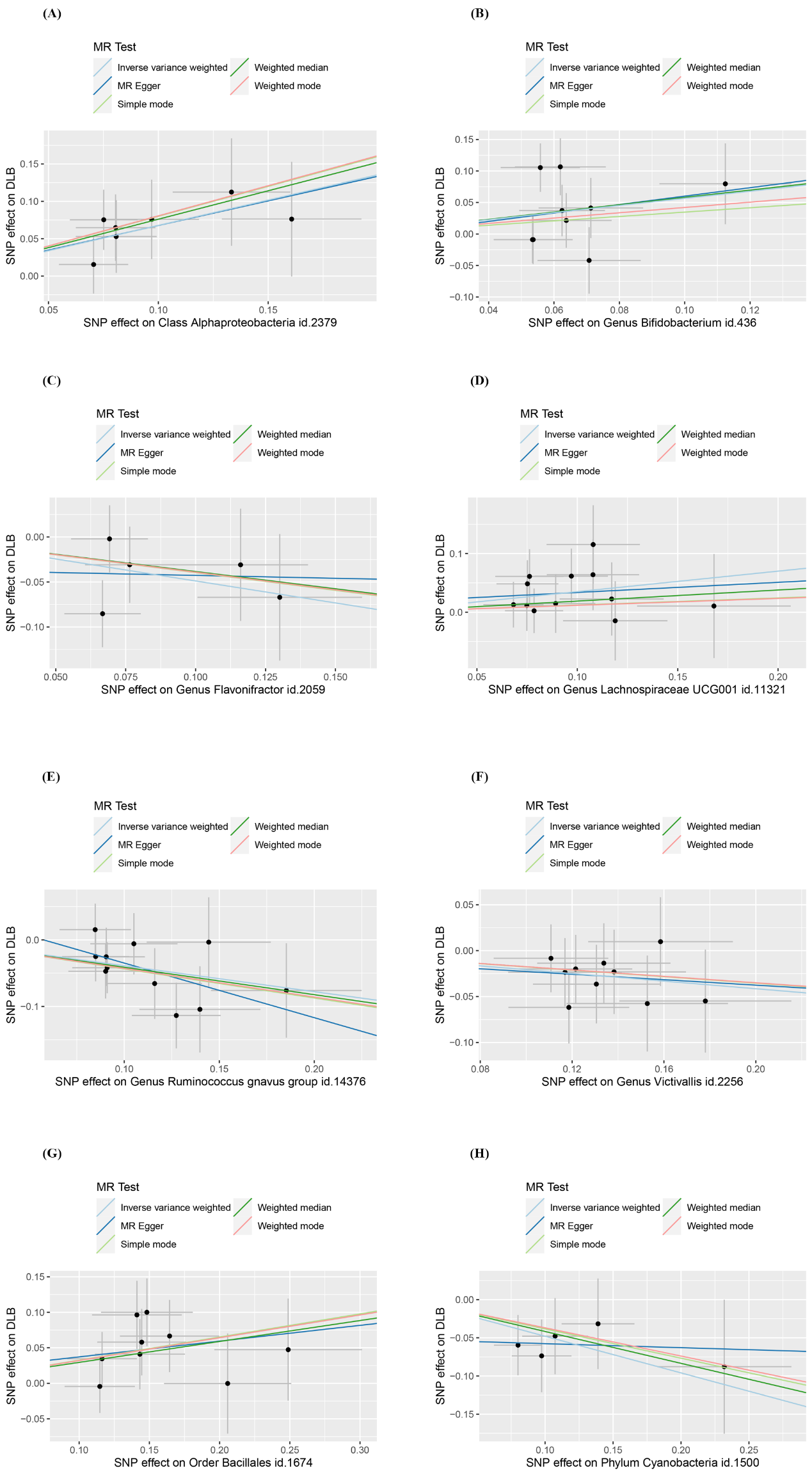


**Figure S9.** Scatter plots for the effect of Gut microbiota on VD.


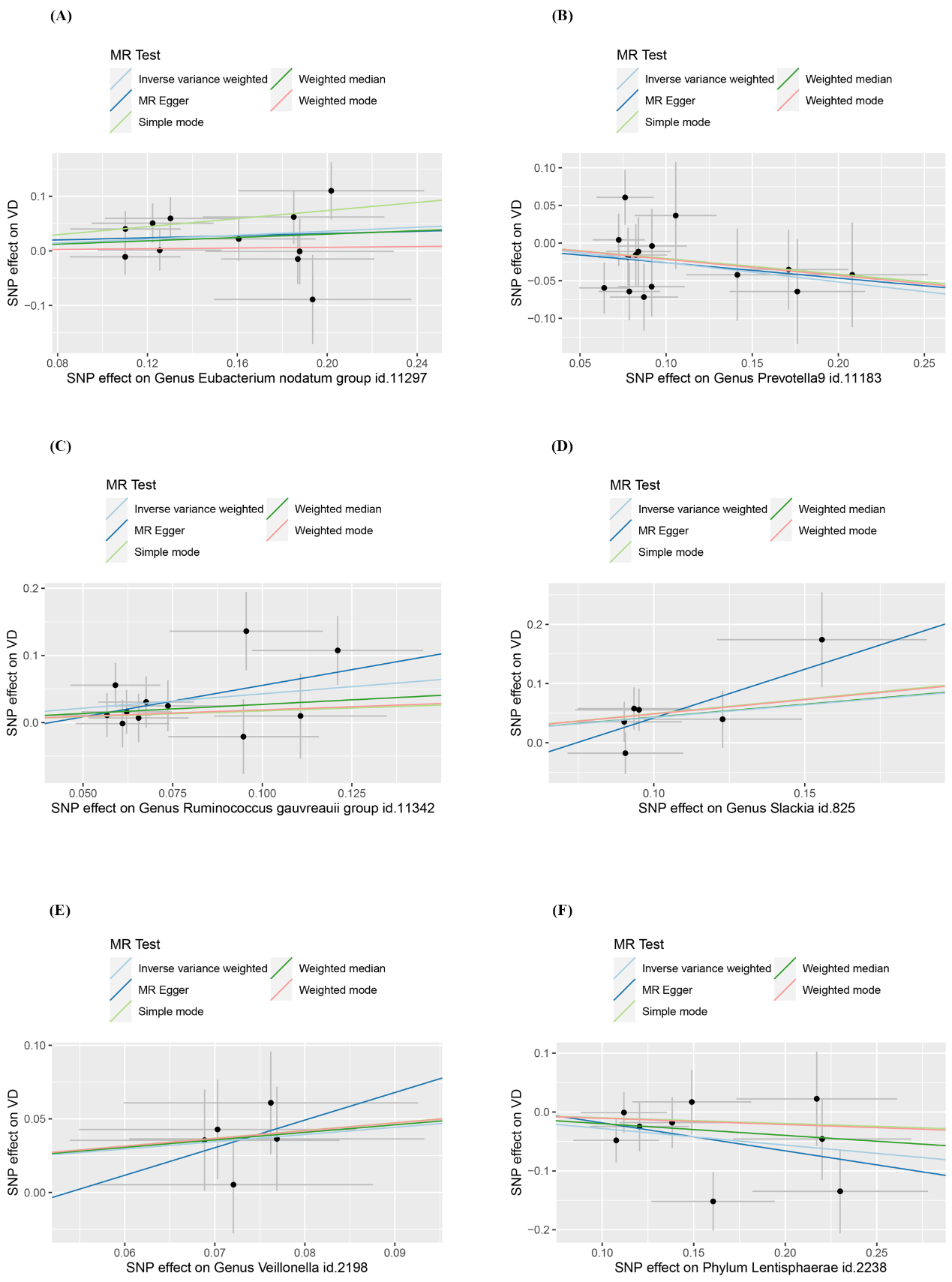


**Figure S10.** Scatter plots for the effect of Gut microbiota on PDD.
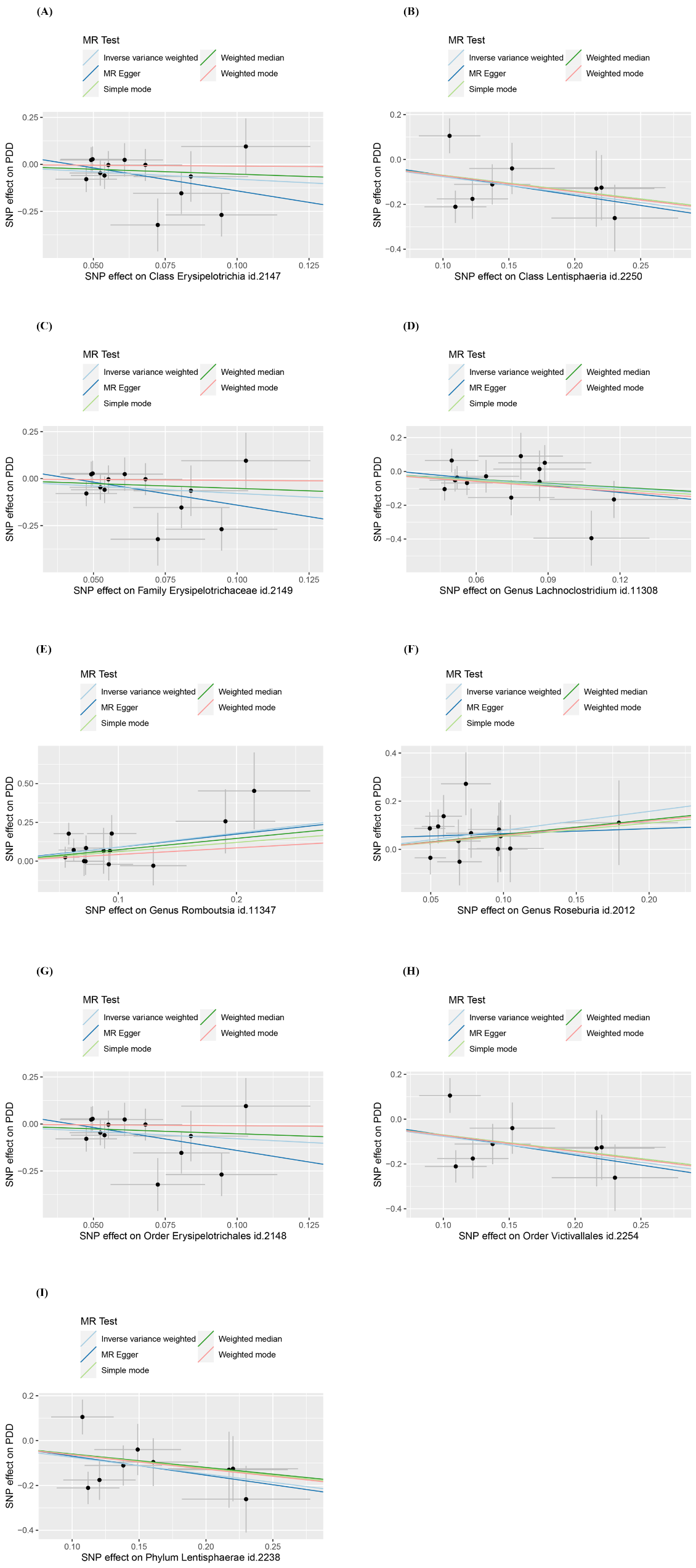


**Figure S11.** Forest plots for the effect of Gut microbiota on AD.


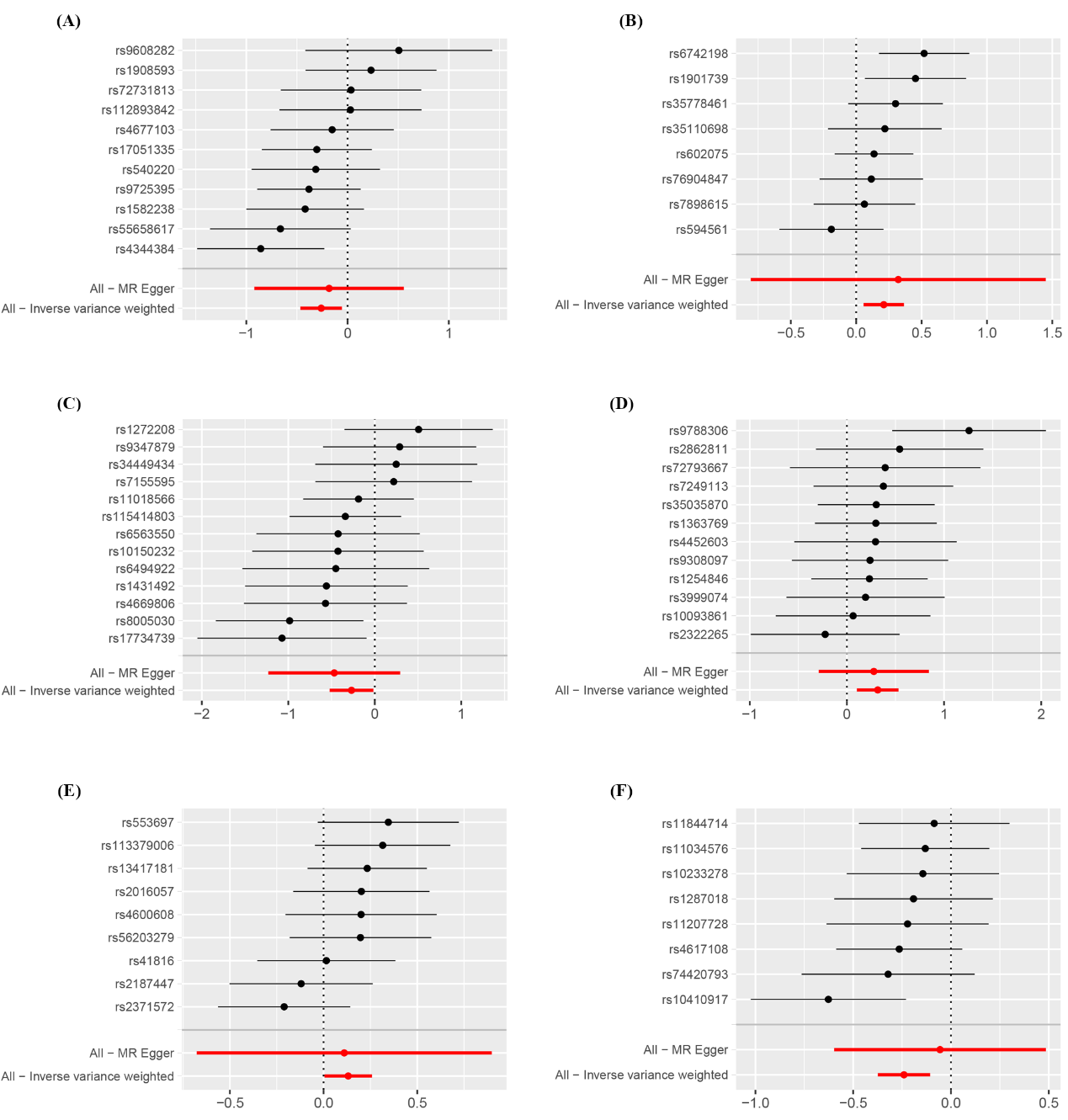


(A) MR effect size for "Family Defluviitaleaceae id.1924" on "AD"

(B) MR effect size for "Genus Allisonella id.2174" on "AD"

(C) MR effect size for "Genus Anaerotruncus id.2054" on "AD"

(D) MR effect size for "Genus Lachnospiraceae FCS020 group id.11314" on "AD"

(E) MR effect size for "Genus Sellimonas id.14369" on "AD"

(F) MR effect size for "Order Bacillales id.1674" on "AD"

**Figure S12.** Forest plots for the effect of Gut microbiota on FTD.


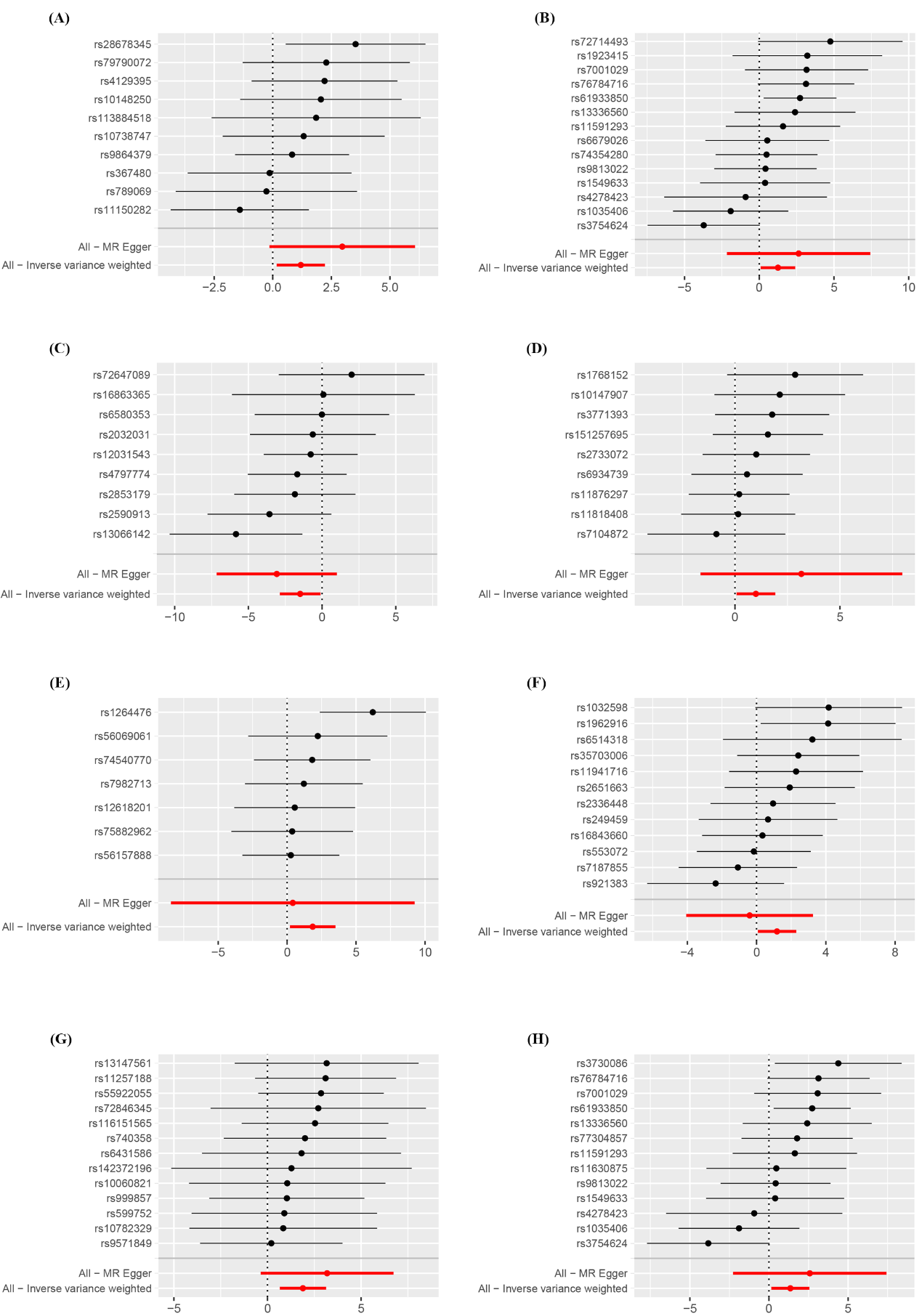


(A) MR effect size for "Class Melainabacteria id.1589" on "FTD"

(B) MR effect size for "Family Rhodospirillaceae id.2717" on "FTD"

(C) MR effect size for "Genus Desulfovibrio id.3173" on "FTD"

(D) MR effect size for "Genus Eubacterium fissicatena group id.14373" on "FTD"

(E) MR effect size for "Genus Phascolarctobacterium id.2168" on "FTD"

(F) MR effect size for "Unknown genus id.2041" on "FTD"

(G) MR effect size for "Unknown genus id.826" on "FTD"

(H) MR effect size for "Order Rhodospirillales id.2667" on "FTD"

**Figure S13.** Forest plots for the effect of Gut microbiota on DLB.


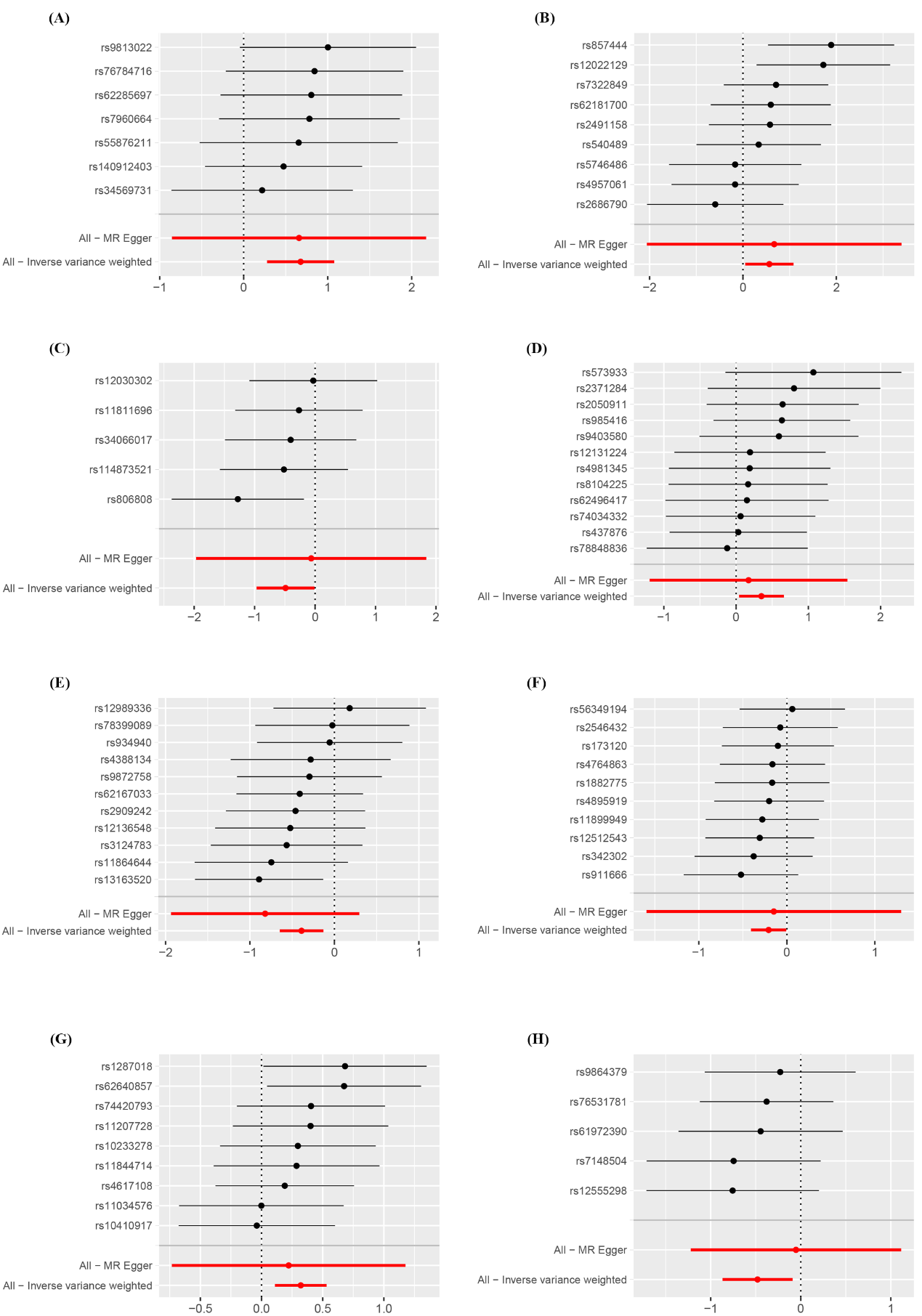


(A) MR effect size for "Class Alphaproteobacteria id.2379" on "DLB"

(B) MR effect size for "Genus Bifidobacterium id.436" on "DLB"

(C) MR effect size for "Genus Flavonifractor id.2059" on "DLB"

(D) MR effect size for "Genus Lachnospiraceae UCG001 id.11321" on "DLB"

(E) MR effect size for "Genus Ruminococcus gnavus group id.14376" on "DLB"

(F) MR effect size for "Genus Victivallis id.2256" on "DLB"

(G) MR effect size for "Order Bacillales id.1674" on "DLB"

(H) MR effect size for "Phylum Cyanobacteria id.1500" on "DLB"

**Figure S14.** Forest plots for the effect of Gut microbiota on VD.


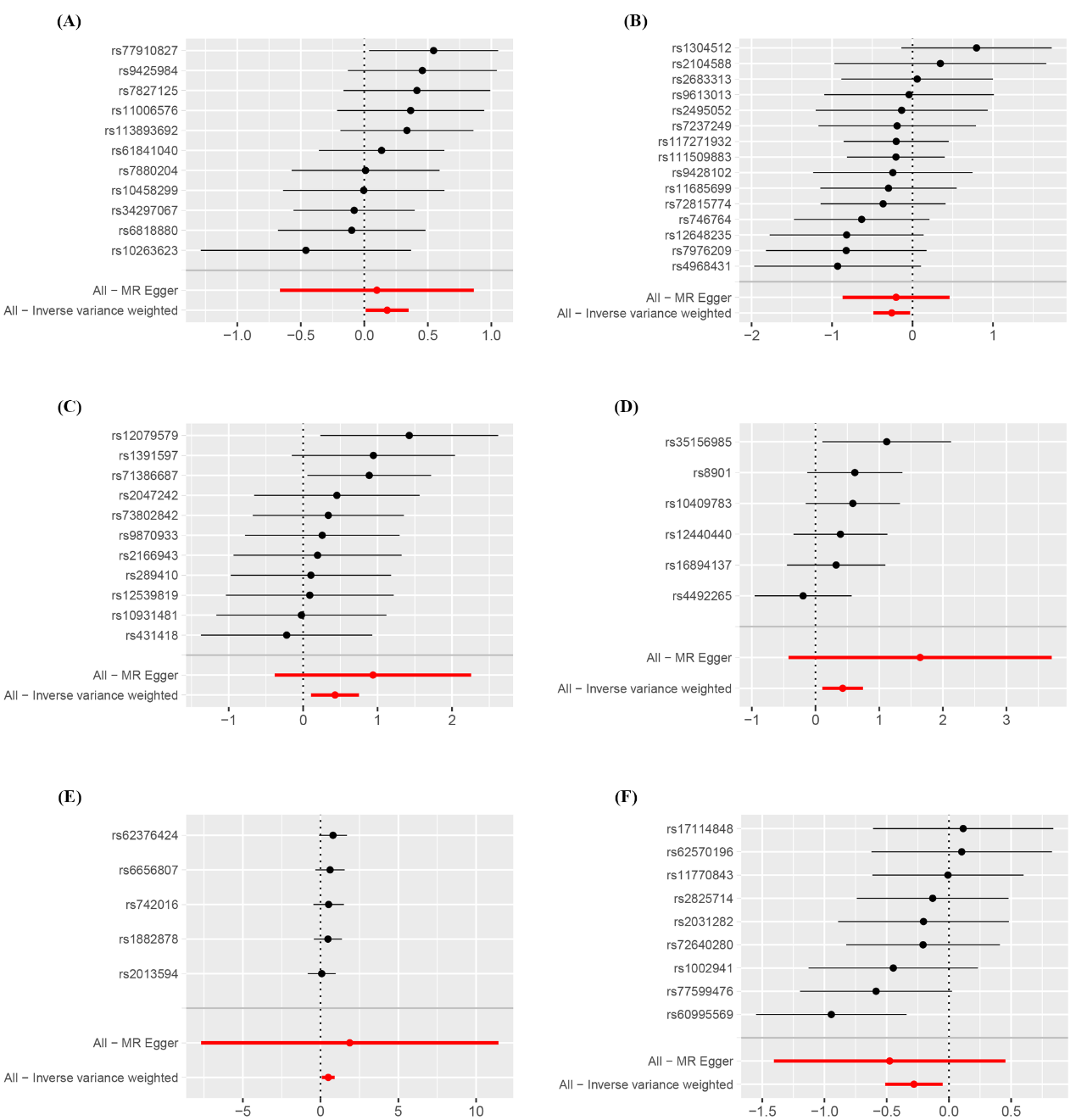


(A) MR effect size for "Genus Eubacterium nodatum group id.11297" on "VD"

(B) MR effect size for "Genus Prevotella9 id.11183" on "VD"

(C) MR effect size for "Genus Ruminococcus gauvreauii group id.11342" on "VD"

(D) MR effect size for "Genus Slackia id.825" on "VD"

(E) MR effect size for "Genus Veillonella id.2198" on "VD"

(F) MR effect size for "Phylum Lentisphaerae id.2238" on "VD"

**Figure S15.** Forest plots for the effect of Gut microbiota on PDD.


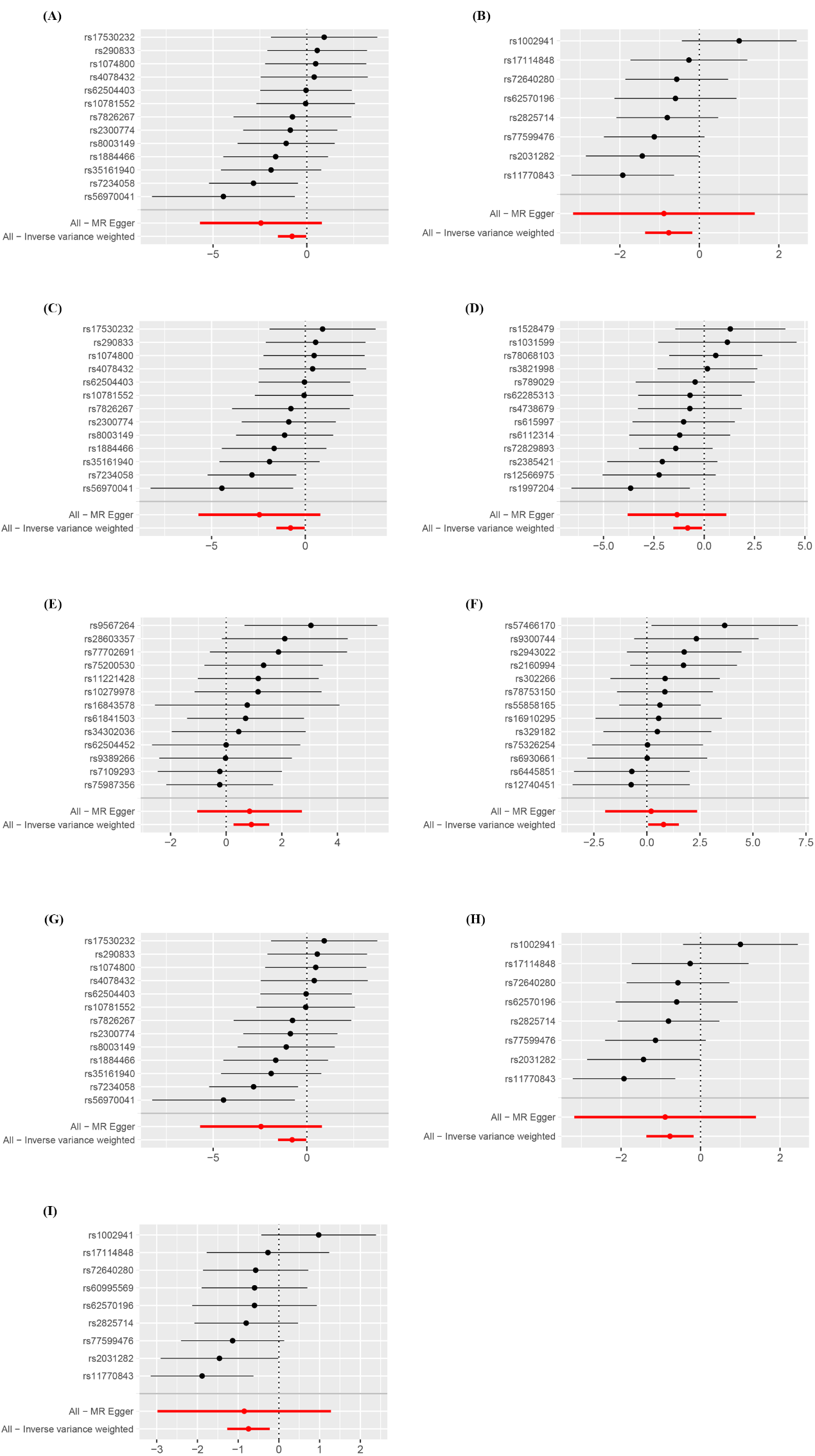


(A) MR effect size for "Class Erysipelotrichia id.2147" on "PDD"

(B) MR effect size for "Class Lentisphaeria id.2250" on "PDD"

(C) MR effect size for "Family Erysipelotrichaceae id.2149" on "PDD"

(D) MR effect size for "Genus Lachnoclostridium id.11308" on "PDD"

(E) MR effect size for "Genus Romboutsia id.11347" on "PDD"

(F) MR effect size for "Genus Roseburia id.2012" on "PDD"

(G) MR effect size for "Order Erysipelotrichales id.2148" on "PDD"

(H) MR effect size for "Order Victivallales id.2254" on "PDD"

(I) MR effect size for "Phylum Lentisphaerae id.2238" on "PDD"
